# Supplementary figures and images for: The Fibrinogen-like Domain of ANGPTL3 Facilitates Lipolysis in 3T3-L1 Cells by Activating the Intracellular Erk Pathway
Source: Biomolecules. 2022 Apr 16;12(4):585. doi: 10.3390/biom12040585 (PMC9028860; doi:10.3390/biom12040585)

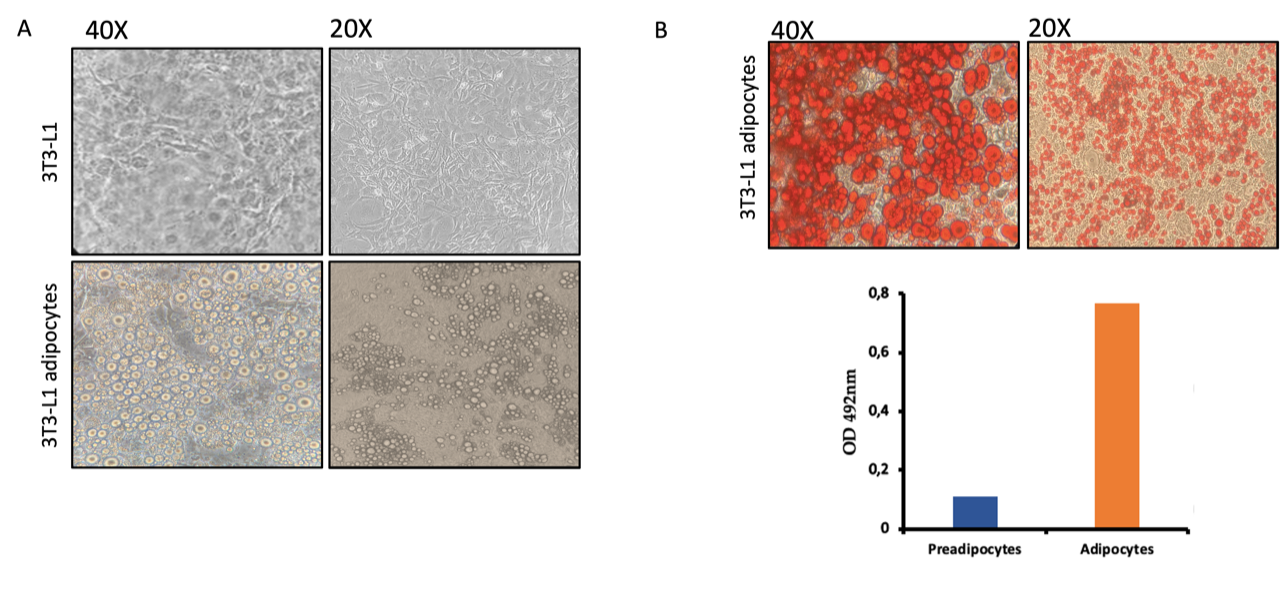

Supplement: Supplementary file 1 [file biomolecules-12-00585-s001.zip › SuppFigure1.png]
